# Comparison of [^18^F]FDG PET/CT with magnetic resonance imaging for the assessment of human brown adipose tissue activity

**DOI:** 10.1186/s13550-020-00665-7

**Published:** 2020-07-22

**Authors:** Jonas Gabriel William Fischer, Claudia Irene Maushart, Anton S. Becker, Julian Müller, Philipp Madoerin, Alin Chirindel, Damian Wild, Edwin E. G. W. ter Voert, Oliver Bieri, Irene Burger, Matthias Johannes Betz

**Affiliations:** 1 Department of Endocrinology, Diabetes and Metabolism, University Hospital Basel, Petersgraben 4, 4031 Basel, Switzerland, and University of Basel, Basel, Switzerland; 2 Institute of Diagnostic and Interventional Radiology, University Hospital Zürich, Rämistrasse 100, 8091 Zürich, Switzerland; 3 Department of Nuclear Medicine, University Hospital Zürich, Rämistrasse 100, Zürich, 8091 Switzerland; 4 Department of Radiology, Division of Radiological Physics, University Hospital Basel, Basel, Switzerland; 5 Division of Nuclear Medicine, University Hospital Basel, Petersgraben 4, 4031 Basel, Switzerland; 6 Department of Radiology, University Hospital of Basel and University of Basel, Basel, Switzerland

## Abstract

**Background:**

Brown adipose tissue (BAT) is a thermogenic tissue which can generate heat in response to mild cold exposure. As it constitutes a promising target in the fight against obesity, we need reliable techniques to quantify its activity in response to therapeutic interventions. The current standard for the quantification of BAT activity is [^18^F]FDG PET/CT. Various sequences in magnetic resonance imaging (MRI), including those measuring its relative fat content (fat fraction), have been proposed and evaluated in small proof-of-principle studies, showing diverging results. Here, we systematically compare the predictive value of adipose tissue fat fraction measured by MRI to the results of [^18^F]FDG PET/CT.

**Methods:**

We analyzed the diagnostic reliability of MRI measured fat fraction (FF) for the estimation of human BAT activity in two cohorts of healthy volunteers participating in two prospective clinical trials (NCT03189511, NCT03269747). In both cohorts, BAT activity was stimulated by mild cold exposure. In cohort 1, we performed [^18^F]FDG PET/MRI; in cohort 2, we used [^18^F]FDG PET/CT followed by MRI. Fat fraction was determined by 2-point Dixon and 6-point Dixon measurement, respectively. Fat fraction values were compared to SUV_mean_ in the corresponding tissue depot by simple linear regression.

**Results:**

In total, 33 male participants with a mean age of 23.9 years and a mean BMI of 22.8 kg/m^2^ were recruited. In 32 participants, active BAT was visible. On an intra-individual level, FF was significantly lower in high-SUV areas compared to low-SUV areas (cohort 1: *p* < 0.0001 and cohort 2: *p* = 0.0002). The FF of the supraclavicular adipose tissue depot was inversely related to its metabolic activity (SUVmean) in both cohorts (cohort 1: *R*^2^ = 0.18, *p* = 0.09 and cohort 2: *R*^2^ = 0.42, *p* = 0.009).

**Conclusion:**

MRI FF explains only about 40% of the variation in BAT glucose uptake. Thus, it can currently not be used to substitute [^18^F] FDG PET-based imaging for quantification of BAT activity.

**Trial registration:**

ClinicalTrials.gov. NCT03189511, registered on June 17, 2017, actual study start date was on May 31, 2017, retrospectively registered. NCT03269747, registered on September 01, 2017.

## Introduction

Brown adipose tissue (BAT) is a thermogenic tissue which contributes to energy homeostasis in human adults. Upon cold exposure, it is activated by the sympathetic nervous system (SNS) and converts chemical energy stored as lipids within the adipocytes directly into heat [1]. Brown adipocytes differ significantly from white adipocytes: they contain a high amount of mitochondria and the intracellular triglycerides are stored in multiple small lipid droplets allowing for rapid lipolysis. The mitochondria in brown adipocytes express high levels of uncoupling protein 1 (UCP1) which is unique to this cell type [2]. When activated by fatty acids, UCP1 allows protons to flow across the inner mitochondrial membrane along the proton gradient which has been built up by the respiratory chain. This is partly uncoupled from ATP synthase. As the proton-motive force driving the ATP synthase is reduced higher amounts of ADP accumulate in turn activating the citric-acid cycle and the respiratory chain. Thus, the activation of UCP1 reduces the efficiency of oxidative phosphorylation and energy stored in the mitochondrial proton-gradient is dissipated as heat [3]. Activation of thermogenic adipocytes increases energy expenditure (EE) and facilitates uptake of glucose and lipids into the tissue [4]. Therefore, BAT is an appealing potential therapeutic target for treatment of obesity and associated metabolic diseases. Since the increase of EE results upon cold exposure, the raise in EE is called cold-induced thermogenesis (CIT). CIT can be determined by measuring the difference EE during warm and cold conditions using indirect calorimetry [5]. In order to reliably evaluate interventions that target energy homeostasis and BAT activity, accurate techniques for quantification are needed. Brown adipocytes are found in the cervical, supraclavicular, axillary, and retroperitoneal region with the cervical and supraclavicular regions being the most predominant [6]. In these adipose tissue depots, thermogenic adipocytes can emerge from white adipocytes in response to cold stimulation. This lineage of brown adipocytes is different from the classical BAT found in human newborns. Upon cold stimulation, these brown-like (brown in white, brite) adipocytes transdifferentiate from white adipose tissue (WAT) [7]. This transitional process from white to brown is highly dynamic, since conversion can be reverted when cold stimulus disappears [8]. This plasticity of the tissue can also be observed in humans in temperate climate zones in whom the amount of active BAT is considerably higher during the cold season [9–11].

Currently, BAT activity is most accurately quantified by [^18^F]fluoro-2-deoxy-D-glucose ([^18^F]FDG FDG) positron emission tomography/computed tomography (PET/CT) as it allows to quantify and visualize and precisely localize metabolically active BAT. However, [^18^F]FDG PET/CT has several limitations: since the radiolabeled tracer [^18^F]FDG PET/CT is taken up preferentially by metabolically active tissue, BAT has to be activated prior to PET/CT scanning. Furthermore, it exposes individuals to ionizing radiation and is expensive [12]. Magnetic resonance imaging (MRI) has been proposed as an alternative to PET/CT [13]. The imaging properties differ significantly from those of WAT since BAT contains more mitochondria and therefore a higher amount of iron and a lower amount of fat [14]. Therefore, the fat fraction (FF) of BAT is generally lower than in WAT. Recent MRI studies described an inverse correlation of the tissues FF and the metabolic activity in [^18^F]FDG PET/CT [13]. However, other studies could not find such correlations [15–18].

In this study, we aimed to quantify the predictive value of MRI FF for BAT by comparing it to the current standard imaging method which is [^18^F]FDG PET/CT.

## Materials and methods

### Study participants

Data from healthy male volunteers participating in two prospective clinical trials (cohort 1: NCT03189511, cohort 2: NCT03269747) was analyzed for this study.

**Fig. 1:**
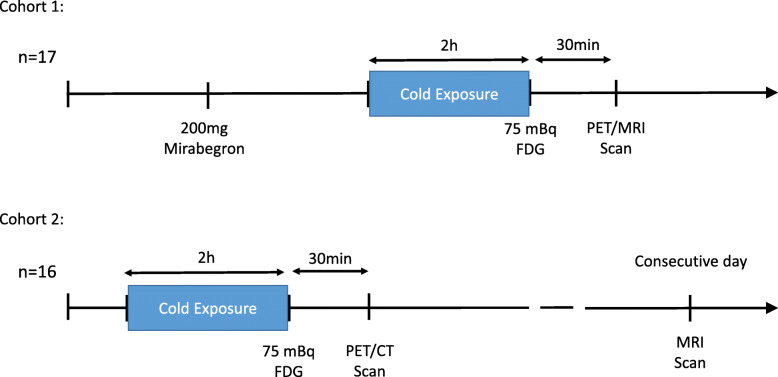
Schematic overview of study procedures in cohorts 1 and 2

Both studies were approved by the Ethics Committee for Northwestern and Central Switzerland (EKNZ). In- and exclusion criteria were identical for both studies. We recruited male volunteers with a BMI between 19 and 27 kg/m^2^ and an age between 18 and 40 years. Exclusion criteria were concomitant disease such as heart-, kidney-, or liver failure, thyroid hormone disorders, hypersensitivity to cold (e.g., Raynaud’s syndrome), and contraindications for MRI. Participants in cohort 1 underwent PET/MRI scanning after 2 h of cold stimulation and 200 mg of the β3-agonist Mirabegron. Participants in cohort 2 underwent PET-CT scanning after a cold stimulus of 2-h duration and an MRI scan on the following day. The study flow for both cohorts is given in Fig. 1. We used a screening visit to identify subjects with an amount of cold-induced thermogenesis (CIT) of at least 5% after a cold stimulus of 2 h.

### BAT stimulation protocols and image acquisition

#### Cohort 1

Participants arrived at the clinical research facility at 8 AM in a fasted state. After measurement of vital signs and body weight, an i.v. cannula was placed in an antecubital vein and blood was sampled. Then participants received 200 mg of Mirabegron. After 90 min, a controlled cold exposure was started using a Hilotherm cooling device (Hilotherm, Argenbühl, Germany) as described previously [19]. Participants wore a T-shirt and shorts during the cooling process. Total cold exposure lasted 120 min. Immediately after cooling, an i.v. bolus of 75 MBq of [^18^F]FDG was applied and static PET image acquisition was performed 30 min later on a GE Medical Signa 3 T PET/MRI Scanner. Images were acquired of the neck/shoulder area using a multi-echo gradient-echo imaging protocol with a monopolar readout. Image acquisition lasted 38 min, including a 30-min dynamic scan followed by a two-bed-position head to upper abdomen scan with a frame time of 4 min each. PET images were reconstructed using 3D ordered subset expectation maximization (OSEM) with 2 iterations and 28 subsets, using a post-reconstruction filter using an in-plane Gaussian convolution kernel with a full-width-at-half-maximum of 5.0 mm, followed by a standard axial filter with a three-slice kernel using relative weights of 1:4:1 [20]. The water and fat MRAC (Magnetic Resonance Attenuation Corrected) series were used to calculate fat fraction images (FF volume) with Matlab with the Signal Processing Package (7.5 R2007b, Mathworks, Natick, MA). The MRI protocol parameters are given in Table 1.

**Table 1:** MRI protocol parameters for cohorts 1 and 2

Protocol name	MR-protocol cohort 1	MR-protocol cohort 2
Description	3D volumetric	3D volumetric
	Interpolated two point	Interpolated multi-echo
	Gradient-echo sequence	Gradient-echo sequence
Echo times (ms)	TE1 = 1.1 (out-of-phase), TE2 = 2.2 (in-phase)	TE1 = 1.09, TE2 = 3.19, TE3 = 5.29, TE4 = 7.39, TE5 = 9.49, TE6 = 11.59
Repetition time (ms)	4	13.5
Flip angle (°)	5	6
Field of view (mm)	500 × 500	256 (read) × 384 (phase)
Image size (voxels)	256 × 256 × 256	192 × 288 × 72
Voxel size (mm)	2.0 × 2.0 × 2.6	1.3 × 1.3 × 1.3
Slice thickness (mm)	2.6	1.3
Field of view phase direction (%)	75	150
Pixel bandwidth (Hz/Px)	1302	810
Slices per slab	120 (per bed position)	72
Acquisition time (min)	0:18 (per bed position)	6:26

#### Cohort 2

Participants arrived fasted at the clinical research facility at 8 AM. After measurement of vital signs and body weight, an i.v. cannula was placed in an antecubital vein and blood was sampled. Baseline calorimetry was performed at warm conditions lasting 30 min. Thereafter, a controlled cold exposure was started using a Hilotherm cooling device (Hilotherm, Argenbühl, Germany) as described previously [19]. Total cold exposure lasted 120 min and indirect calorimetry was performed during the last 30 min of cooling. Immediately after cooling, an i.v. bolus of 75 MBq of [^18^F]FDG was applied and static PET image acquisition was performed 30 min later on a Biograph mCT PET/CT Scanner (Siemens Healthineers, Erlangen, Germany). In order to reduce the exposure to ionizing radiation, low-dose CT scanning was limited to two-bed positions starting from the lower margin of the orbitae thus covering the supraclavicular and axillary fat depots. Low-dose CT (120 kVp 20–100 mAs) was performed with a 3-mm slice thickness and FOV of 500 mm. CT reconstruction was done with a Siemens proprietary algorithm (I30F medium smooth). Attenuation corrected PET was reconstructed with TOF, 2 iterations 21 subsets and a Gaussian filter of 5 mm. Co-registered images were reviewed in 3-orthogonal planes.

MRI was performed 1 day after the PET scanning on a Magnetom Prisma 3 T scanner (Siemens, Erlangen, Germany). Images were acquired of the neck/shoulder area using a 3D multi-echo gradient-echo imaging protocol with monopolar readout. Image acquisition lasted 6:26 min. Participants wore a T-shirt and shorts. The MRI protocol parameters are given in Table 1. FF was calculated in Matlab with the Signal Processing Package (Mathworks, Natick, MA) using the mDixon-separation method. A graphical representation of the study procedures is given in Fig. 1.

### Image analysis

We analyzed the resulting 3d volumes using the 3D Slicer software, version 4.10.1 (National Institutes of Health, Bethesda, MD) [21].

**Fig. 2:**
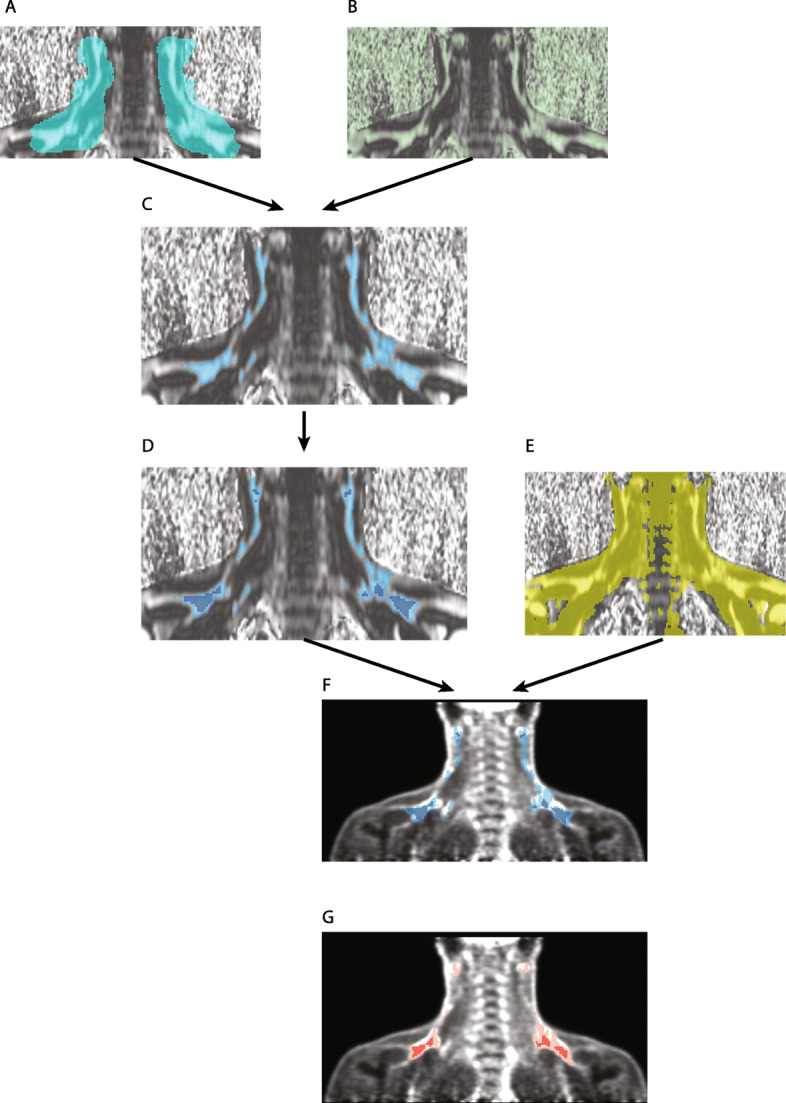
Image processing workflow. **a** Segmentation based on a crude manual outline of the adipose tissue in the neck region. **b** Segmentation based on thresholding of the fat fraction volume selecting all voxels with a fat fraction between 400 and 1000 per mille. **c** Intersection of segmentations **a** and **b** leading to an exact representation of the adipose tissue in the neck region. **d** This segmentation was shrunk by one voxel in each dimension in order to reduce partial volume effects. **e** To reduce background noise the in-phase volume was thresholded in order to select the contours of the body and intersected with segmentation **d** resulting in a final segmentation **f** (overlay with PET data). This segmentation was again intersected with areas within the PET data volume exhibiting SUV values above 1.5 g/ml yielding a representation of the metabolically active supraclavicular adipose tissue

To establish a predictive model of MRI FF for PET activity, we used a distinct segmentation approach (see segmentation flow in Sup. Fig. 1), the single steps of which are outlined in Fig. 2. Briefly, segmentation was based on (1) a crude anatomic delineation of the supraclavicular adipose tissue depot, (2) thresholding based on the FF in the MRI volume, and (3) the [^18^F]FDG uptake in PET. By using the sphere brush tool of 3D slicer, the adipose tissue in the neck/shoulder area was roughly outlined (Anatomical segmentation, Fig. 2a). Second, we used the FF volume and included all voxels with a FF of 400–1000‰ as further segmentation (FF segmentation, Fig. 2b). Next, we intersected these segmentations to generate the region of interest comprising the adipose tissue within the supraclavicular region (Fig. 2c). In order to reduce partial volume effects, we scaled the segmentation down by one voxel in each dimension using the margin/shrink tool of 3D slicer (Fig. 2d). In order to reduce noise, we used a threshold on the in-phase volume (Fig. 2w), i.e., masking voxels outside the boundaries of the subject’s body. In cohort 1, MRI and PET volumes were co-registered (PET/MRI). Accordingly, the corresponding PET image could be superimposed (Fig. 2f). SUV and FF values were quantified in the respective data volumes using the segmentations resulting from the workflow described above.

In a second approach, we intersected the already established FF segmentation with a segmentation based on the PET volume using a threshold of SUV ≥ 1.5 (g/ml). The resulting segmentation comprises the adipose tissue with the highest metabolic activity (Fig. 2g).

#### PET/CT data (cohort 2)

We used a similar segmentation approach as described above. First, by using the sphere brush tool of 3D slicer, the adipose tissue in the neck/shoulder area was roughly segmented (Anatomical Segmentation). Second, we used the CT image series and included all voxels within the range of − 190 and −10 HU in a second segmentation (radiodensity segmentation). The third step included a segmentation with voxels exhibiting a SUV ≥ 1.5 (g/ml) (PET segmentation) according to the BARCIST 1.0 recommendation [12]. Then, we intersected the three segmentations resulting in a final segmentation. As in cohort 1, we reduced the dimensions of the segmentation by one voxel in each dimension using the margin/shrink tool of 3D slicer (optimized final segmentation). Finally, we quantified the [^18^F]FDG uptake values by using the final segmentation and optimized reduced segmentation on the PET image series.

#### Intra-individual differences in fat fraction

**Fig. 3:**
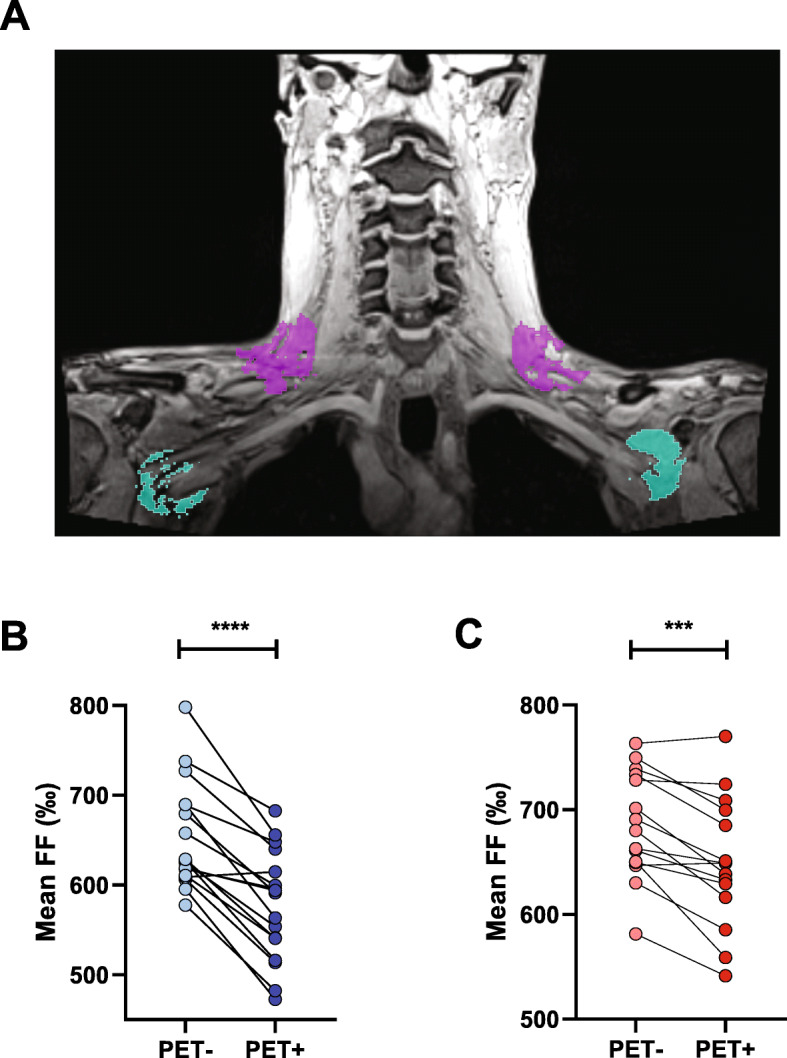
Comparison of the fat fraction in PET-negative and PET-positive areas within the supraclavicular adipose tissue depot. **a** Placement of spherical region of interest in PET-negative (turquois) and PET-positive (pink) area of supraclavicular adipose tissue. **b** Study cohort 1, *p* < 0.0001 and **c** study cohort 2, *p* = 0.0002 (paired *t* test).

In order to compare the FF between PET-positive and PET-negative areas within the supraclavicular adipose tissue depot, we placed a sphere in the region of the highest FDG uptake as well as in a region without discernable [^18^F]FDG uptake. The spheres were placed using the sphere brush tool (diameter = 5%) and were intersected with the segmentation containing metabolically active BAT (Fig. 3a).

### Statistical analysis

Data is presented as mean ± standard deviation, unless otherwise stated. Statistical analysis was performed and figures were created using GraphPad Prism v. 8.2.1 (San Diego, CA) for linear regression to predict BAT activity in PET/MRI FF. Pairwise comparisons were performed with paired *t* tests. A *p* value below 0.05 was considered to indicate significance. To enhance prediction, a multiple linear regression model to estimate SUV_mean_ was used in which age, BMI, and CIT were included as contributing independent factors. These calculations were performed with the R statistical software [22] and the nlme package [23].

## Results

### Participant characteristics

A total of 17 healthy male volunteers, 23.4 ± 3.2 years old, participated in cohort 1. Their average BMI was 23.4 ± 1.7 kg/m^2^. One of the participants (age 22 and BMI 24.7 kg/m^2^) did not exhibit active BAT after mild cold exposure and was therefore excluded from the analysis.

Cohort 2 consisted of 16 healthy male volunteers aged 24.5 ± 4.4 years with a BMI of 22.5 ± 2.3 kg/m^2^.

### Within depot variance of fat fraction in comparison to local metabolic activity

The metabolic activity as assessed by [^18^F]FDG uptake varies considerably within the supraclavicular adipose tissue (scAT) depot leading to areas which are PET-positive (high [^18^F]FDG uptake) and others which are PET-negative (very low [^18^F]FDG uptake). We assessed the FF of the scAT in each individual in the PET-positive areas and the corresponding PET-negative areas bilaterally (Fig. 3a). In line with the notion of lower fat content in the metabolically active, thermogenic adipose tissue, FF in the PET-negative area of the supraclavicular adipose tissue depot of cohort 1 was 653 ± 59؉ as compared to 577 ± 61؉ in the BAT-positive area (*p* < 0.0001, Fig. 3b). In cohort 2, the FF in the PET-negative area was 685 ± 51؉ as compared to 649 ± 62؉ in the BAT-positive area (*p* < 0.0002, Fig. 3c).

### Relation of supraclavicular adipose tissue fat fraction to BAT activity

**Fig. 4:**
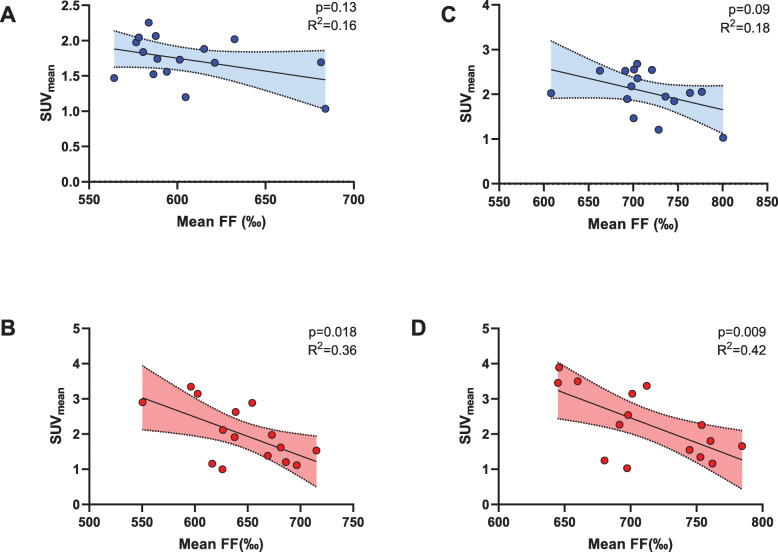
Simple linear regression plots with SUV_mean_ (g/ml) as dependent variable versus tissue fat fraction (‰) as the independent variable. Cohort 1, final segmentation (**a**) and the more restrictive optimized final segmentation (**b**). Cohort 2, final segmentation (**c**) and the more restrictive optimized final segmentation (**d**)

Next, we determined whether the FF in the supraclavicular adipose tissue depot was significantly related to its [^18^F]FDG uptake. In cohort 1, FF of the complete scAT depot (FF_sc-total_) was inversely related to SUV_mean_, but this association did not reach statistical significance (*R*^2^ = 0.16, *p* = 0.13, Fig. 4a). In cohort 2, we observed a statistically significant inverse relation of FF_sc-total_ and SUV_mean_ (*R*^2^ = 0.36, *p* = 0.018, Fig. 4b).

In order to avoid influence from partial volume effects, we reduced the size of the segmentation by one voxel in each dimension. This slightly improved the predictive accuracy for both cohorts (cohort 1: *R*^2^ = 0.18, *p* = 0.09 and cohort 2: *R*^2^ = 0.42, *p* = 0.009, Fig. 4 c and d).

### Fat fraction in PET-positive supraclavicular adipose tissue is related to the metabolic activity

**Fig. 5:**
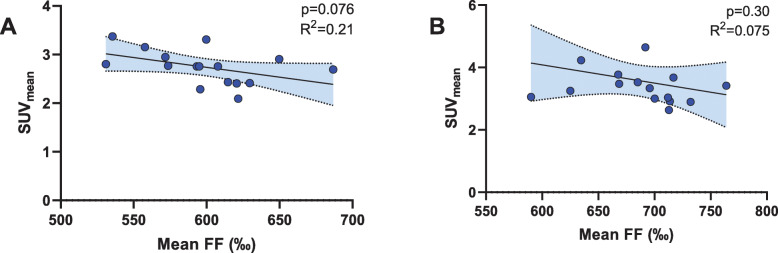
Simple linear regression plots with SUV_mean_ (g/ml) as dependent variable versus tissue fat fraction (‰) as the independent variable

Using co-registered PET/MRI scans in cohort 1 allowed us to measure FF selectively in areas with high activity in PET (SUV_mean_ ≥ 1.5 g/ml). While the correlation was slightly better than that for FF_SC-total_, it was not statistically significant (*R*^2^ = 0.21; *p* = 0.076). Shrinking the segmentation volume to avoid partial volume effects worsened predictive performance (*R*^2^ = 0.075; *p* = 0.3), as shown in Fig. 5. This might be due to the fact that the resulting segmentation was relatively small.

### Relation of BAT activity at the core of supraclavicular adipose tissue to fat fraction

Human BAT shows a cranio-caudal gradient of activation [6] with the supraclavicular depot being universally present if active BAT is found. Moreover, the peak of FDG uptake within the scAT depot is often found close to the supraclavicular fossa. We therefore speculated that measuring the FF at this location might be helpful to predict BAT activity, especially without prior knowledge of the tissue’s activity in FDG-PET. However, the measurement of FF at this region did not correlate significantly to the corresponding FDG uptake (cohort 1: *R*^2^ = 0.04, *p* = 0.47; cohort 2: *R*^2^ = 0.15, *p* = 0.15).

### Relation of fat fraction and [^18^F]FDG uptake in both cohorts

In order to be able to analyze the pooled results from both cohorts, we performed z-scaling of the scalar values followed by simple linear regression of FF_total_ vs. [^18^F]FDG uptake. In this pooled analysis (*n* = 32), we found a significant correlation between the two values (adjusted *R*^2^ = 0.26, *p* = 0.018).

### Relation of cold-induced thermogenesis to adipose tissue fat fraction and BAT in [^18^F]FDG PET/CT

**Fig. 6:**
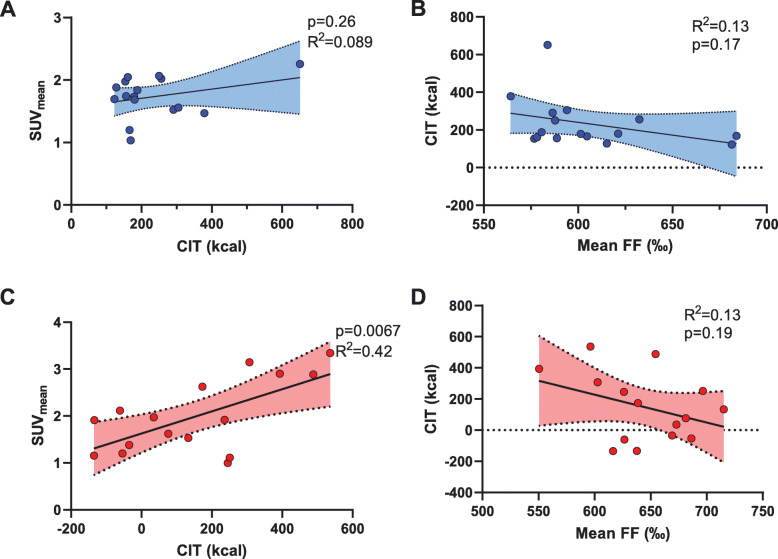
Linear regression of FDG uptake versus cold-induced thermogenesis (CIT) and fat fraction versus CIT in cohort 1 (**a** and **b**) and cohort 2 (**c** and **d**)

In cohort 1, we assessed cold-induced thermogenesis (CIT) in each individual by subtracting the RMR under warm conditions from the RMR under cold conditions 7 to 14 days prior to measuring BAT activity in [^18^F]FDG PET/CT. FDG uptake into BAT was not significantly associated to CIT (*R*^2^ = 0.09, *p* = 0.26) which might be due to the fact that CIT was not measured on the same day as [^18^F]FDG uptake. Also, the FF in this cohort was not statistically significant related to CIT (*R*^2^ = 0.13, *p* = 0.17) (Fig. 6a and b). In cohort 2, we measured CIT directly prior to the PET/CT scanning. [^18^F]FDG-uptake into BAT was significantly related to CIT (*R*^2^ = 0.42, *p* = 0.0067). The FF_SC-total_ in the same cohort was only weakly associated with CIT (*R*^2^ = 0.13, *p* = 0.19), as shown in Fig. 6c and d.

### Multiple linear regression for prediction of [^18^F]FDG uptake

In order to establish a more robust prediction model for [^18^F]FDG uptake, we pooled the data from our two cohorts and performed a multiple linear regression model after z-scaling with FF, age BMI, and CIT (see Table 2). For cohort 1, prediction was weak (adjusted *R*^2^ = 0.07, *p* = 0.33). For cohort 2 however, prediction was strong (adjusted *R*^2^ = 0.58, *p* = 0.01). Combining both cohorts SUV_mean_ was significantly predictable (adjusted *R*^2^ = 0.33, *p* = 0.0055).

**Table 2:** Contributions of selected parameters to the multiple linear regression models

Cohort 1					Cohort 2			
Parameter	Estimate	Std. error	*t* value	*p*	Estimate	Std. error	*t* value	*p*
FF	− 0.3647	0.2628	− 1.388	0.193	− 0.3407	0.2327	− 1.464	0.1739
Age	− 0.2353	0.2684	− 0.877	0.399	− 0.3288	0.2116	− 1.554	0.1512
BMI	− 0.2454	0.2657	− 0.924	0.375	0.2371	0.2347	1.01	0.3362
CIT	0.02787	0.2725	0.102	0.92	0.7118	0.2404	2.961	0.0143

## Discussion

In the present study, we evaluated whether measurement of the fat fraction (FF) within the supraclavicular fat depot (scAT) in vivo can reliably predict the metabolic activity of brown adipose tissue (BAT) as assessed by [^18^F]FDG PET/CT. Within each individual, the FF differed significantly between the PET-negative and PET-positive area of the scAT which is in line with the notion that human BAT is highly heterogeneous, consisting of brown (thermogenic) and white adipocytes and that highly active BAT contains a higher amount of mitochondria and less fat [8]. However, the predictive power of the FF in the scAT depots was relatively low, accounting only for 16 to 36% of the variance in BAT activity. Previous studies in this field had already evaluated the relation of BAT FF and its metabolic activity in smaller cohorts or as proof-of-principle studies. While some studies demonstrated a relatively good correlation between MRI determined FF and BAT activity, others did not find significant associations.

In a group of 13 healthy volunteers, Holstila et al. found a good association between glucose uptake and FF both during cold exposure and warm ambient conditions [13]. Remarkably, the measured FF varied substantially among individuals and FF of WAT and BAT depots were even sometimes within the same range. FF, as measured in this study, accounted for approximately 40% in the variation of FDG uptake. In a similar study including ten healthy individuals from the same research group, FF was shown to predict BAT activity (*R*^2^ = 0.41, *p* = 0.04) [24]. In a cohort of twelve healthy volunteers studied by [^18^F]FDG PET/MRI, the authors found a good correlation between FF and BAT glucose uptake rate in cold-stimulated supraclavicular BAT (*R*^2^ = 0.52) [25]. A study in 20 healthy volunteers using cold exposure and capsinoid administration to activate the SNS assessed different imaging modalities and found a significant correlation (*R*^2^ = 0.39 *p* = 0.012) between SUV_mean_ and FF [26].

However, other research groups using a similar approach could not find a significant relation between MRI measured FF and BAT activity as assessed by [^18^F]FDG PET/CT [18]. Likewise, a larger study including 28 children and adolescents evaluated MRI parameter changes in respect to BAT activity measured by [^18^F]FDG PET/CT [27]. Using voxel-wise co-registration, they were not able to find any direct correlation between FF and BAT activity as assessed by [^18^F]FDG-PET under different thermal conditions. Nevertheless, they could show that changes in FF from cold to re-warming correlated with BAT activity. A retrospective study analyzed MRI and [^18^F]FDG-PET in 66 pediatric patients to determine whether MR imaging can reproducibly detect human BAT independent of its activation state. The authors did not find a statistically significant and sufficiently predictive correlation of SUV_mean_ with FF and hypothesized that this might be due to relative stable FF and highly variable SUV_mean_ values [17].

Some of the differences in study outcome might be explained by different methodological approaches. First, the determination of FF by MRI is complex and multi-echo sequences might be more suitable than dual-echo sequences. This could theoretically explain the different outcomes from our cohorts 1 (dual-echo) and 2 (multi-echo). However, a recent study demonstrated similar results for dual-echo and multi-echo sequences [25].

Second, controversy exists whether to use glucose uptake rate (GUR) and dynamic PET scanning versus standardized uptake values (SUV) and static PET scanning as primary PET/CT readout. One should take into consideration that BAT GUR is less sensitive to confounders such as body weight and meal intake [12, 28]. However, in the setting of fasted individuals after a defined cold stimulus GUR and SUV_mean_ seem to correlate very well [29].

Third, the segmentation approach can also influence the quantification of FF mainly due to partial volume effects that can significantly affect the FF. We tried to reduce this effect by shrinking the segmentation as a final step before quantification [30]. Moreover, it remains an open question which part of the scAT depot should be segmented. Segmentation of the whole depot might underestimate the amount of thermogenically active BAT. On the other hand, segmentation based on the post hoc knowledge of the highest [^18^F]FDG uptake is not an option if MRI-based techniques should substitute [^18^F]FDG-PET for evaluation of BAT activity. The studies which could show a robust inverse correlation between BAT activity in PET/CT and FF in MRI often placed the FF measurements into the area of BAT with the highest glucose uptake [13, 15, 24]. Another study found a significant correlation between FF and glucose uptake only in a subgroup with highly active BAT but failed to do so in individuals with lower GUR [24].

Since we were interested in developing a predictive tool, we segmented the scAT depot in MRI without prior knowledge of PET results. While our approach resulted in a moderate correlation of FF and BAT activity, the coefficient of determination (*R*^2^) was significantly lower. This is in line with other studies, which based their segmentation on the whole scAT depot [17, 18, 27]. It should be noted, however, that Anderson et al. had a quite good predictive result using this strategy [25].

Nevertheless, a more likely explanation for the relatively low predictability might be that the differences in scAT FF are not merely due to the amount of thermogenic adipocytes or the mitochondrial density of the tissue. Our results and data from previous studies [13] indicate that the inter-individual variability of the FF among the participants is large, ranging from 577.6 to 798‰ in cohort 1 and 581.2 to 763.4‰ in cohort 2. Within the same cohort, we found an average absolute difference of 76‰ and 35‰ respectively of intensity in areas of high SUV activity compared to areas of lower SUV activity. This means that the intra-individual differences in FF between thermogenically active and inactive fat are smaller than the inter-individual range. This might be due to the fact that BAT FF content is affected by age [31] and body weight [27]. Additionally, the FF in subcutaneous thermogenically inactive adipose tissue is also variable and decreases during cold exposure indicating lipolysis [24]. This is underscored by the fact that a recent study was not able to establish a threshold to reliably detect BAT by using FF in MRI [32]. Also, another explanation could be that leaner and younger subjects who generally possess more active BAT have lower FF in their adipose tissue depots.

It should be noted that, [^18^F]FDG PET/CT measures glucose uptake in to BAT and not thermogenic activity as such. The determination of oxygen consumption and blood flow in the tissue have been evaluated previously, but are technically much more challenging [33]. From a physiological point of view, cold-induced thermogenesis (CIT) seems to be a more important variable to measure. BAT activity correlates with CIT but does not fully determine this phenomenon as probably muscle contributes substantially to CIT also [34]. Another newly discovered mechanism adding to CIT might be a proton leak in tissues such as skeletal muscle. It is mediated by ATP/ADP-cotransporter, working as a parallel proton channel besides of UCP1 [35]. Therefore, multiple determinants of SUV_mean_ might be necessary to be taken into consideration, e.g., CIT, age, BMI, and FF. Using multiple linear regression of these parameters in cohort 2 in which we measured CIT directly before PET/CT, we could reach an improved predictive value of *R*^2^ = 0.58. We think this strategy of integrating FF, age, BMI, and CIT to predict BAT activity may be sufficiently accurate for the use in prospective cohort studies or as a screening tool.

Our study is limited in several aspects: first, we used static PET imaging. Several authors propose instead to measure GUR in dynamic PET scanning. This technique is less prone to errors due to [^18^F]FDG PET/CT uptake into muscle [34] rather than performing static PET and measure SUV_mean_. Still, as measurements were only performed on fastened individuals, glucose uptake in muscle is minimized and SUV_mean_ correlated very well with BAT activity [29]. Second, our study included only young healthy male participants, making the cohort homogenous but not reflecting the general population. Third, the BAT activation protocols differed in the fact that we used a β3 agonist in cohort 1 in addition to controlled cold exposure. Strengths of our study comprise the relatively large sample size of 32 participants, a systematic analysis of the imaging data which was unbiased by prior knowledge of the PET signal as well as the combined analysis of MRI FF and CIT.

## Conclusion

Taken together, MRI determined FF clearly separated metabolically active from inactive adipose tissue within the scAT depot of an individual. However, FF could not predict BAT activity within a cohort or across different cohorts with a high degree of accuracy. Using a multi-echo Dixon sequence in cohort 2, we achieved a coefficient of determination (*R*^2^) of 0.4 which is in line with other published studies. A coefficient of determination within a range of *R*^2^ = 0.4 to 0.5 which will preclude the use of MRI FF measurement for the quantitative assessment of BAT activity, e.g., in the context of a clinical trial. To improve prediction, it may be used in combination with different measurements such as CIT and anthropometric data and might be suitable for screening purposes. Currently, the mainstay of BAT quantitative imaging will remain [^18^F]FDG-PET/CT or [^18^F]FDG-PET/MRI.

## Supplementary information

**Additional file 1: Supplementary Figure 1.** Segmentation Flow for cohort 1 and cohort 2.

## Data Availability

The datasets used and/or analyzed during the current study are available from the corresponding author on reasonable request. The request will be judged by an independent committee at the Department of Clinical Research of the University Hospital Basel to ensure that legal obligations are fulfilled.
